# Infiltration and Water Use Efficiency of Maize Fields with Drip Irrigation and Biodegradable Mulches in the West Liaohe Plain, China

**DOI:** 10.3390/plants12050975

**Published:** 2023-02-21

**Authors:** Yayang Feng, Haibin Shi, Yanhui Jia, Qingfeng Miao, Qiong Jia, Ning Wang

**Affiliations:** 1 College of Water Conservancy and Civil Engineering, Mongolia Agricultural University, Huhhot 010018, China; 2 Farmland Irrigation Research Institute, Chinese Academy of Agricultural Sciences (CAAS), Xinxiang 453002, China; 3 High-Efficiency Water-Saving Technical Equipment and Water and Soil Environmental Effects Inner Mongolia Autonomous Region Engineering Research Center, Huhhot 010018, China; 4 Inner Mongolia Institute of Hydraulic Research, Huhhot 010051, China

**Keywords:** drip irrigation, biodegradable mulch, precipitation infiltration, yield, water use efficiency

## Abstract

Biodegradable mulches have the same temperature- and moisture-preservation effects as ordinary plastic mulches before degradation. After degradation, rainwater enters the soil through the damaged parts, improving precipitation utilization. Under drip irrigation with mulching, this study explores precipitation utilization of biodegradable mulches under different precipitation intensities and the effects of different biodegradable mulches on the yield and water use efficiency (WUE) of spring maize in the West Liaohe Plain, China. In this paper, in situ field observation experiments were conducted for three consecutive years from 2016 to 2018. Three types of white degradable mulch films were set up, with induction periods of 60 d (WM60), 80 d (WM80), and 100 d (WM100). Three types of black degradable mulch films were also used, with induction periods of 60 d (BM60), 80 d (BM80), and 100 d (BM100). Precipitation utilization, yield, and WUE under biodegradable mulches were studied, with ordinary plastic mulches (PM) and bare land (CK) set as controls. The results showed that as precipitation increased, the effective infiltration of precipitation decreased first and then increased. When precipitation reached 89.21 mm, plastic film mulching no longer affected precipitation utilization. Under the same precipitation intensity, the precipitation effective infiltration ratio increased as the damage to the biodegradable film increased. Still, the intensity of this increase gradually decreased as the damage increased. The highest yield and WUE were observed for the degradable mulch film with an induction period of 60 days in years with normal rainfall and for the degradable mulch film with an induction period of 100 days in dry years. In the West Liaohe Plain, maize planted under film receives drip irrigation. We recommend that growers select a degradable mulch film with a degradation rate of 36.64% and an induction period of approximately 60 days in years with normal rainfall, and a degradable mulch film with an induction period of 100 days in dry years.

## 1. Introduction

The West Liaohe Plain is located in the northeast China corn belt, one of the three largest corn belts worldwide [1]. According to meteorological observation data of Tongliao City from 1983 to 2016, the annual average precipitation in the plain was 337.2 mm; precipitation is mostly distributed between July and August, accounting for 51.09% of the annual precipitation [2]. These characteristics have been attributed to global warming and the accompanying increase in the frequency of low spring temperatures in northeast China [3]. Low temperatures and uneven precipitation in the spring pose severe challenges to the growth of spring maize in the West Liaohe Plain. Mulching is a planting method used to reduce the impact of low spring temperatures on maize [4]. However, ordinary plastic mulches (PM) contribute to “white pollution” and present increasingly prominent problems [5], such as obstructing rainwater utilization in the middle and late growth stages [6]. Mulching can substantially improve crop growth in the early growth stage but it excessively consumes soil nutrients [7,8].

Using biodegradable mulch can alleviate the negative impacts of mulching [9,10,11]. By adding a degradation aid to traditional plastics, biodegradable mulch quickly degrades hydrophobic macromolecules into small hydrophilic molecules under light, heat, oxygen, water, and other environmental factors. Digested and absorbed by microorganisms, mulches finally degrade to carbon dioxide, water, and humus. Before degradation, biodegradable mulches have the same temperature-increasing and moisture-retaining effects as PM [12,13,14]. After degradation, the warming effect is reduced, which can decrease the negative impact on yield attributed to excessive soil temperature caused by PM in the middle and later stages of crop growth [15,16,17]. Upon degradation, rainwater can infiltrate the crop root zone through the damaged parts of the mulch, thereby increasing precipitation utilization. Chen [16] analyzed the impact of precipitation on the moisture content of different mulch treatments, and the biodegradable mulch treatment increased precipitation utilization efficiency by 8.2% compared with the common mulch treatment. Feng [18] reported that the difference in soil moisture between biodegradable mulch and PM treatments occurred mainly within 0–20 cm, and the soil moisture content of the PM treatment was 2.53–3.01% lower than that of biodegradable mulch treatment.

Most studies have found no significant difference in yield and water use efficiency (WUE) between biodegradable mulch and PM treatments [19,20,21,22]. Other studies have also pointed out that the yield under PM treatment is the highest; however, biodegradable mulch treatments exhibited no significant difference in WUE. Wang [23] found that compared with no mulching, biodegradable mulches achieved better performance in enhancing cotton yield and WUE in the dry year (916kg/ha;1.85kg/mm·ha) than in the wet year (568kg/ha;1.22kg/mm·ha). Yin [13] conducted three consecutive years of maize experiments from 2014 to 2016 and showed that the average yield and WUE of the B2 treatment with a medium degradation rate were significantly higher than those of the common mulch and degradable mulches with faster (B1) and slower (B3) degradation rates. They reported that although the precipitation intensity was higher in 2014 than in 2015, the yield and WUE of each treatment were higher in 2015 than in 2014. Therefore, in addition to the degradation rate, the intensity and distribution of rainfall during the maize growing season can also affect crop yield and WUE.

Most studies on biodegradable mulches focus on the soil environment, crop growth indicators, and yield, and the reported impacts on yield differ according to regional and climate characteristics. There are few studies on precipitation utilization under biodegradable mulches, and most focus on comparing soil moisture content. To the best of our knowledge, there are no in-depth studies on the characteristics of precipitation infiltration under different precipitation intensities. Because the upper boundary conditions of biodegradable mulches change with time and precipitation cannot be controlled, research on this topic is complex and dynamic. There are also few studies on the applicability of biodegradable mulches for the typical climate conditions of the West Liaohe Plain, with a large gap in research on the characteristics of rainwater infiltration under different precipitation intensities upon the use of biodegradable mulches.

Biodegradable mulches in drip irrigation can be effectively used to address the typical climate characteristics of the West Liaohe Plain and the severe overexploitation of groundwater. The main objective of this study is to investigate the regular pattern of precipitation infiltration under biodegradable mulches and evaluate the precipitation effective infiltration ratio under different precipitation intensities. Biodegradable mulches suitable for additional hydrological years were prioritized to increase crop yield and WUE. The results are expected to provide a reasonable basis for promoting and applying biodegradable mulches in the West Liaohe Plain to maintain the environment’s safety and food supplies.

## 2. Results

### 2.1. Degradation Rates of Biodegradable Mulches

**Figure 1:**
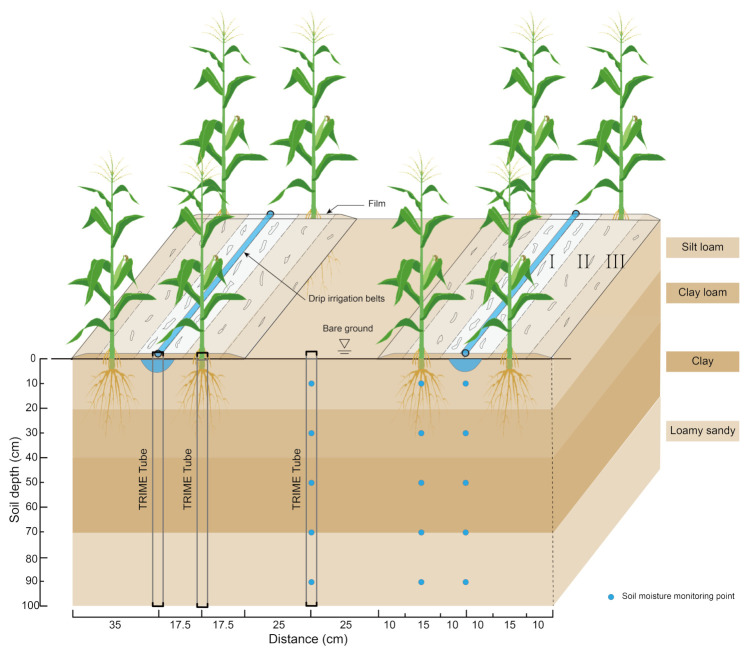
Schematic diagram of planting mode, soil moisture monitoring, and degradation membrane breakage.

**Figure 2:**
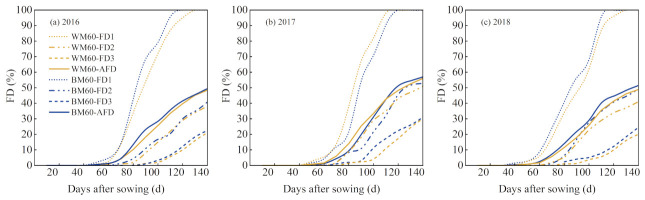
Degradation rates of biodegradable mulches in Zones I, II, and III. WM60, white degradable mulch films with induction periods of 60 d. BM60, black degradable mulch films with induction periods of 60 d.

Degradation rates of biodegradable mulches in Zones I, II, and III are shown in Figure 1 and Figure 2. After sowing for 50 d, the biodegradable mulches in Zone I began to deteriorate; the degradation speed was accelerated on day 60; and the mulches were completely damaged at approximately day 110. The mulches in Zone II began to deteriorate on day 70, and the degradation rate was slower than that of Zone I. The mulches in Zone III began to deteriorate on day 90, presenting the slowest degradation rate. The degradation rate $FD_{1}$ (fraction of the degradation area of Zones I) of WM60 (white degradable mulch films with induction periods of 60 d) and BM60 (black degradable mulch films with induction periods of 60 d) reached 100% at the end of the growth period, and there was nearly no mulch on the ground surface. The respective degradation rates $FD_{2}$ were 35.26–50.69% and 39.60–52.88%, whereas $FD_{3}$ were 20.14–29.71% and 20.42–30.52%. Zones II and III were covered by shallow soil. Still, the degradation rate of Zone II was higher than that of Zone III because the soil layer covering the former was shallower, and the soil environment fluctuated to a higher extent owing to irrigation, precipitation, and crop growth.

### 2.2. Effects of Different Film Mulching on Soil Moisture Content before Precipitation

**Figure 3:**
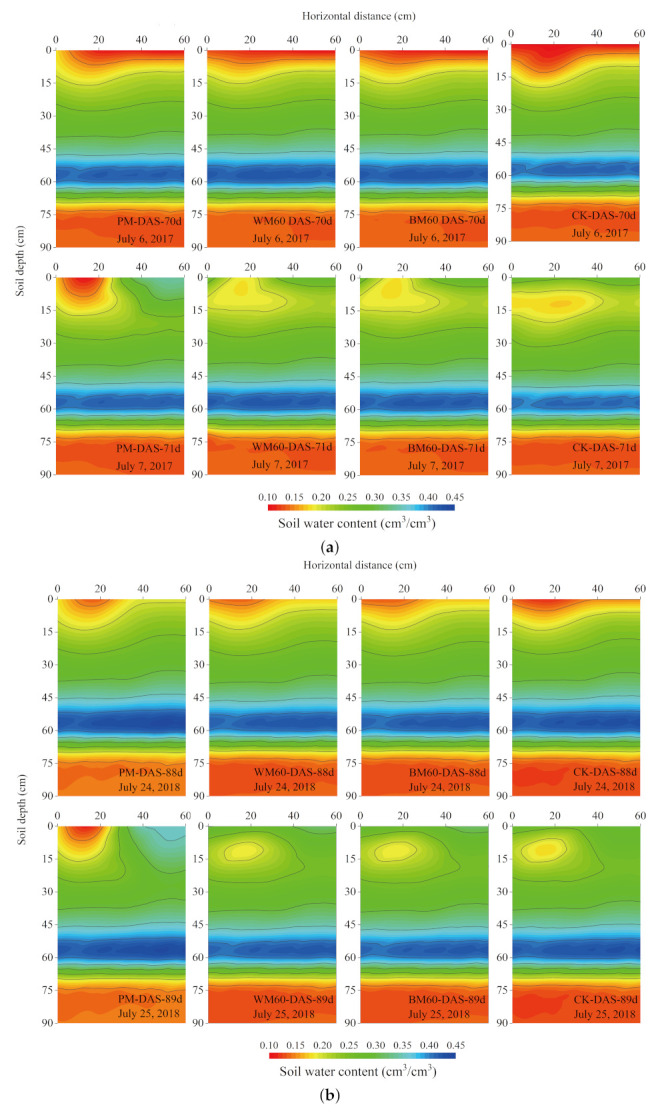

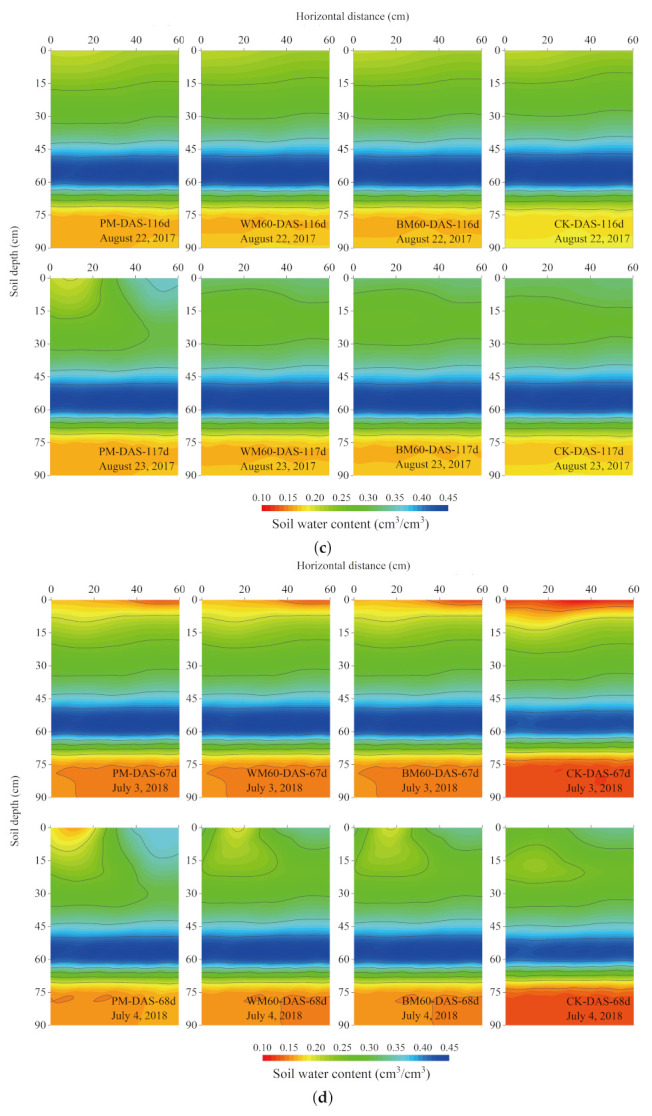

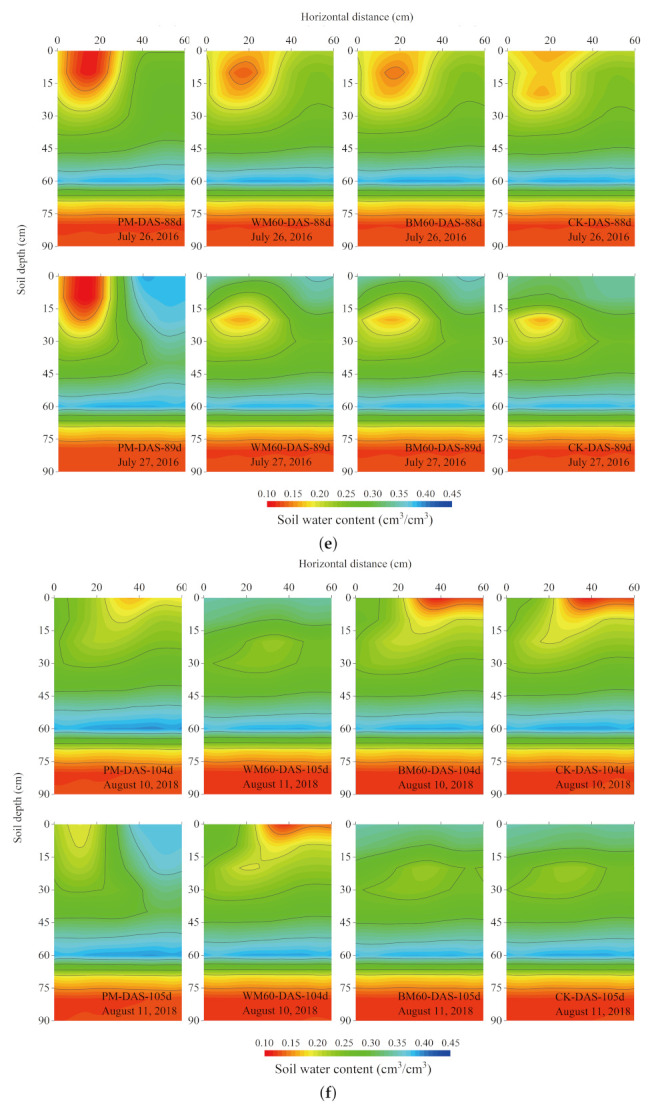

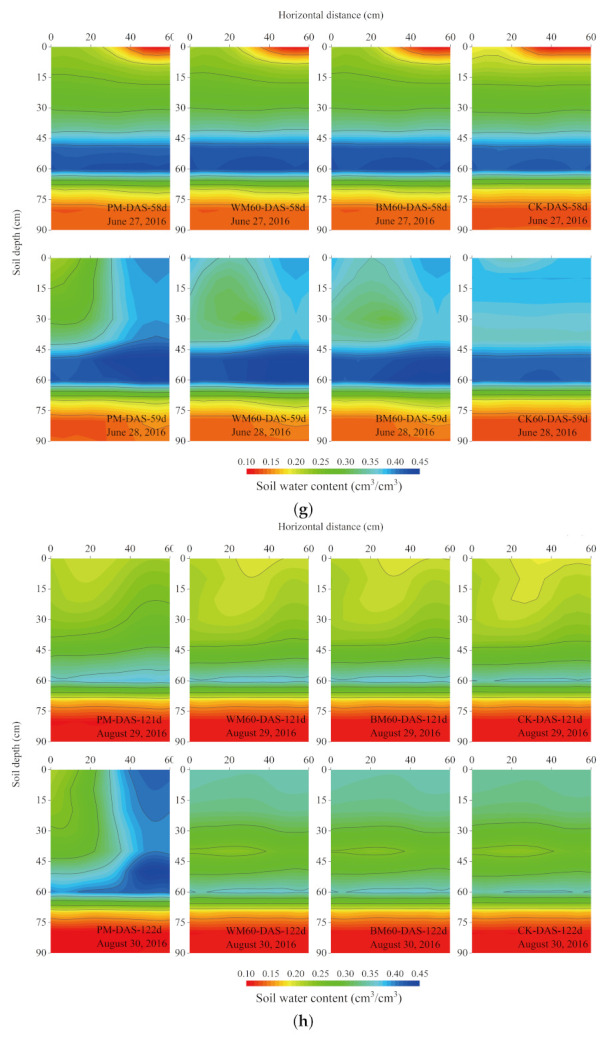
Dynamic change of soil moisture content before and after precipitation treated by different types of mulching. Moderate precipitation: (**a**) 7 July 2017, (**b**) 25 July 2018, (**c**) 23 August 2017. Heavy precipitation: (**d**) 4 July 2018, (**e**) 27 July 2016, (**f**) 11 August 2018. Extremely heavy precipitation: (**g**) 28 June 2016, (**h**) 30 August 2016.

Before precipitation, differences between the soil moisture contents upon PM, biodegradable mulch, and CK treatments mainly occurred in the 0–20 cm soil layer. At different stages of maize growth, the soil moisture content in the 0–60 cm soil layer under mulching was higher than that under the CK treatment. Approximately 60 d after sowing (Figure 3a,d,g), when the degradation rate of biodegradable mulches was low, the difference in soil moisture content compared with the PM treatment was insignificant. Approximately 90 d after sowing (Figure 3b,e), the average soil moisture content of the 0–60 cm soil layer under biodegradable mulches was 2.91–4.93% higher than that of PM and the moisture content of the area without mulching was reduced by 3.85–7.62%.

Moreover, during this period, the biodegradable mulches were partially degraded and the rainwater could directly infiltrate through the deteriorated parts. However, the mulches still retained part of the water; so, the soil moisture content in the area with mulching was slightly higher than in the area with PM. Approximately 110 d after sowing (Figure 3c,f,h), the average soil moisture contents of the 0–60 cm soil layer under biodegradable mulches and without mulching were 1.62–3.74% and 7.19–8.93% lower than those of PM, respectively. At this stage, the degradation rate of the biodegradable mulches was relatively high, and the water retention effect of the area with mulching gradually disappeared. In the later maize growth stage with withering and falling leaves, the maize canopy coverage decreased and the evaporation intensity increased, leading to a lower soil moisture content for biodegradable mulches than PM. The moisture content of the soil without biodegradable mulching was lower than that of PM owing to the imperviousness of PM and rainwater runoff into the area without mulching.

### 2.3. Precipitation Infiltration Depth

As precipitation intensity increased, the depth of rainwater penetration increased (Figure 3). At different stages of maize growth, the moisture content of the surface soil in the bare land treatment and area without mulching increased rapidly after precipitation, but this change decreased with increasing soil depth. The changes in soil moisture content in areas with mulching under PM were small and even lower than before precipitation under moderate precipitation conditions. The degradation rate of biodegradable mulches and precipitation infiltration were different, affecting rainwater redistribution.

### 2.4. Precipitation Effective Infiltration Ratio

Under moderate precipitation (Figure 3a–c), as the degradation rate of biodegradable mulches increased, the maximum depth of rainwater infiltration in Zone I under mulching increased from 10 to 15 cm, whereas those in Zones II and III increased from no rainwater infiltration to 10 cm. The maximum infiltration depth in the area without mulching decreased from 25 to 15 cm. As the degradation rate of biodegradable mulches increased, the depth of rainwater infiltration in the area with mulching gradually increased and that in the area without mulching gradually decreased.

Under heavy precipitation, the maximum depth of rainwater infiltration in Zone I (15 cm) was achieved for degradation rates of 7.97–9.82% (Figure 3d), whereas the maximum depths in Zones II and III (10 cm) occurred as the degradation rate increased to 18.81–21.32% (Figure 3e). The maximum depth of rainwater infiltration in areas with mulching did not increase with degradation rate, which suggested that the depth of rainwater infiltration in areas with biodegradable mulching was affected not only by its degradation rate but also by the soil moisture before precipitation. Lower soil moisture content before precipitation led to a shallower soil depth of rainwater infiltration. When the degradation rate increased to 39.03–41.71% (Figure 3f), the maximum depth of rainwater infiltration in Zones I and II under mulching after precipitation was 30 cm, whereas that in Zone III was 20 cm under mulching and without mulching. The infiltration depth of rainwater in the area without mulching was smaller than that with mulching because the soil moisture content in the latter was significantly higher than in the former before precipitation.

Under extremely heavy precipitation, when the degradation rate was 1.97–2.47% (Figure 3g), the maximum depth of rainwater infiltration in Zones I and III under mulching was 20 cm, whereas that in Zone II was 10 cm. The infiltration depth outside the mulching area was 90 cm. When the degradation rate increased to 35.89–37.46% (Figure 3h), the maximum depth of rainwater infiltration was 60 cm, which was not significantly different from that of the CK treatment.

Under moderate precipitation, as the degradation rate of biodegradable mulches gradually increased, the precipitation effective infiltration ratio also gradually increased. At a degradation rate of 9.51–10.28%, the precipitation effective infiltration ratio was 46.05–47.61%. As the degradation rate increased to 22.83–24.71%, the precipitation effective infiltration ratio increased to 50.60–51.09%. When the degradation rate further increased to 39.57–42.48%, the precipitation effective infiltration ratio increased to 51.20–52.41%, which was 51.20–59.72% higher than that of the PM treatment and 6.60–24.13% lower than that of the CK treatment, with significant differences (*p* < 0.05). Under heavy precipitation, the degradation rate was 7.97–9.82% and the precipitation effective infiltration ratio was 30.66–31.64%. The precipitation effective infiltration ratios under PM and CK treatments were 20.61% and 47.71%, respectively, with significant differences (*p* < 0.05). At a degradation rate of 18.81–21.32%, the precipitation effective infiltration ratio was 41.88–42.06%. As the degradation rate increased to 39.03–41.71%, the precipitation effective infiltration ratio decreased to 40.99–41.51%. For the CK treatment, the ratio of precipitation effective infiltration was 43.13%, which was not significantly different from that upon biodegradable mulch treatment. As the degradation rate increased, the precipitation effective infiltration ratio first increased, and after reaching a certain degradation rate, it gradually plateaued.

Under extremely heavy precipitation, the degradation rate of the biodegradable plastic film increased from 1.97–2.47% to 35.89–37.46%. Consequently, the ratio of precipitation effective infiltration increased from 27.06–28.23% to 43.98–44.01%, whereas that of the CK treatment was 47.19%, which was not significantly different from that of biodegradable mulches.

### 2.5. Construction and Analysis of Precipitation Effective Infiltration Ratio Equation

We fitted the data using multiple linear regression to obtain model equations of precipitation (*x*) and degradation rate of mulch (*y*) as follows:

(1) $$\lambda =54.7167-2.2580x+1.4297y+0.0310x^{2}-0.0238y^{2}+0.045xy,R^{2}=0.9591,p<0.01$$

The coefficients of the independent variables in the regression equation indicated that the absolute values of the coefficients of the independent variable *x* (precipitation) were greater than the independent variable *y* (degradation rate). This suggested that the ratio of precipitation effective infiltration was affected by precipitation to a greater extent than the degradation rate. The quadratic term of precipitation was positive, which indicated that as precipitation increased, the precipitation effective infiltration decreased first and then increased. In contrast, the quadratic term of the degradation rate was negative, indicating that the precipitation effective infiltration first increased as the degradation rate increased.

**Figure 4:**
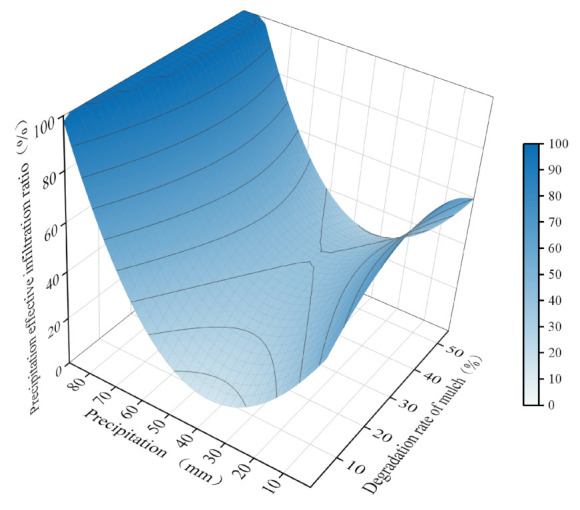
Interaction of precipitation and damage rate on precipitation effective infiltration ratio.

We set the precipitation range to 1–84 mm, its step length to 2.8 mm, the degradation rate to 1–55%, and its step length to 1.8%. We then drew a 3D surface map by substituting these values into the regression equation (Figure 4). The lowest ratio of precipitation effective infiltration occurred when precipitation was 24–37 mm. After precipitation reached 89.21 mm, the mulch no longer affected precipitation utilization in the mulching area. As the degradation rate of biodegradable mulch increased, the precipitation required for the minimum ratio of precipitation effective infiltration gradually decreased. For a degradation rate of 1%, the minimum rate of precipitation effective infiltration corresponded to precipitation of 35.69 mm. As the degradation rate increased to 15.53%, the minimum rate of precipitation effective infiltration corresponded to precipitation of 25 mm. When the degradation rate was 36.64%, the minimum ratio of precipitation effective infiltration corresponded to 10 mm of precipitation. During the 3 y study period, 56 precipitation events occurred—of which only 7 presented precipitation greater than 25 mm—and the West Liaohe Plain experienced mainly light and moderate precipitation. Therefore, the results suggest that the degradation rate of the biodegradable mulches reached 36.64% when the rainy season began.

### 2.6. Yield and WUE

The overall water consumption pattern was consistent over the three study years, and the water consumption by PM-treated crops was the lowest. The largest water consumption upon using biodegradable mulches occurred for the induction period of 100 d, followed by induction periods of 80 and 60 d. The CK treatment led to the largest water consumption and, despite the significant difference between the PM and CK treatments in 2016 (*p* < 0.05), there were no significant differences between the treatments in 2017 and 2018 (*p* > 0.05).

There were no significant differences between the yields of white and black biodegradable mulches for the same induction periods (*p* > 0.05), as well as between PM, WM100, and BM100 treatments. In 2016, the yields of WM60 and WM80 increased by 8.12% and 4.19% compared with WM100, whereas the yields of BM60 and BM80 increased by 7.21% and 4.88% compared with the yield of BM100. In 2017, there were no significant differences between treatments with biodegradable mulches for induction periods of 60 and 80 d. The yields of WM60 and WM80 treatments increased by 5.12% and 0.10% compared with the WM100 yield, whereas BM60 and BM80 treatments increased by 4.37% and 0.08% compared with that of BM100. In normal years, shorter mulching periods led to higher yields. In 2018, the yields of WM60 and WM80 were 12.09% and 2.13% lower than that of WM100, whereas the yields of BM60 and BM80 increased by 8.31% and 1.66% compared with that of BM100. In the dry years, the yields gradually decreased as the induction period increased.

Mulching significantly improved the WUE of the maize crops under the same irrigation level (Table 1). The WUE under mulching and bare land treatments was maintained within 25.41–30.08 and 24.02–28.37 kg/mm·ha, respectively. In 2016 and 2017, the average WUE of WM60, BM60, WM80, BM80, WM100, and BM100 compared with the CK treatment increased by 2.14%, 1.22%, 1.18%, 0.49%, 0.99%, and 0.31%, respectively. There was no significant difference in the WUE under the PM, WM100, BM100, and CK treatments. In 2018, the WUE of the PM treatment (28.77 kg/mm·ha) increased by 10.78%, 14.58%, and 16.98% compared with the WM60, BM60, and CK treatments, respectively. There were no significant differences between the water use efficiencies of WM80, BM80, WM100, and BM100 (*p* > 0.05).

## 3. Discussion

In this study, the moisture content of mulch treatments was higher than that of CK before precipitation. At approximately 60 d after sowing, the biodegradable mulches began to degrade, which was not significantly different from the PM treatment. At approximately 90 d, the biodegradable mulches were partially degraded, yet still provided moisture retention while promoting the utilization of precipitation through the damaged parts. This is consistent with the conclusions of a previous study [16,18]. Liu [24] investigated the distribution of soil moisture content and soil precipitation accumulation for winter maize before and after precipitation under a PM treatment. The results showed that compared with CK, the PM treatment led to higher soil moisture content before precipitation. However, the soil moisture content in the 40–60 cm soil layer was lower than the initial soil moisture content after precipitation, and mulching could not effectively increase the soil moisture after precipitation. Conversely, owing to strong transpiration, the soil moisture in the later stage of crop jointing was relatively low. In this study, similar results were obtained for the PM treatment under moderate precipitation. Biodegradable mulching prevented the scenario in which the moisture content after precipitation was lower than that before precipitation because the rainwater infiltrated directly from the damaged parts.

Song’s research on precipitation infiltration showed that compared with drip irrigation without mulching, the infiltration ratio and wetting front migration ratio of drip irrigation under mulching were both reduced [25]. Moreover, higher precipitation intensity decreased the effect of mulching. This study revealed that as precipitation increased, the depth of rainwater infiltration in the mulched area increased, whereas the precipitation effective infiltration decreased first and then increased. When the precipitation reached 89.21 mm, mulching no longer affected the precipitation utilization in the mulched area. This occurred because rainwater directly infiltrated the soil from the damaged parts under low precipitation. As precipitation increased, the moisture content in the surface soil of the damaged area was saturated. Because the rainwater could not further infiltrate the soil from the damaged parts in time, surface runoff occurred and flowed into areas without mulching, thereby reducing the precipitation effective infiltration ratio. As precipitation continued to increase, the lateral migration of soil moisture content in areas without mulching increased after saturation, thereby gradually increasing the precipitation effective infiltration ratio again.

In this study, under moderate and extremely heavy precipitation, as the degradation rate increased, rainwater infiltration in areas with mulching increased, and the ratio of precipitation effective infiltration also gradually increased. However, the magnitude of this increase gradually decreased with the increase in degradation rate. In contrast, as the degradation rate increased under heavy precipitation, the precipitation effective infiltration first increased and then decreased. As the degradation rate increased from 18.81–21.32% to 39.03–41.71%, the ratio of precipitation effective infiltration decreased from 41.88–42.06% to 40.99–41.51%, which represented a slight decline. This was attributed to the difference in the intensity and distribution of moisture content before precipitation. For a degradation rate of 18.81–21.32%, the moisture content of the mulched area was close to the wilting coefficient, and the soil moisture content of the area without mulching was significantly higher than that of the mulched area. This promoted the direct absorption of rainwater in the mulched area, whereas water in areas without mulching also migrated laterally as the degradation rate reached 39.03–41.71%, and the distribution of moisture content before precipitation was inverted. The soil moisture content in the mulched area was significantly higher than in areas without mulching owing to the irrigation 3 d before precipitation. Therefore, the ratio of precipitation infiltration was related not only to the degradation rate of the mulches and precipitation intensity but also to the intensity and distribution of soil moisture content before precipitation. Lower soil moisture content before precipitation led to a shallower depth of rainwater infiltration and a higher ratio of precipitation effective infiltration. Furthermore, the degradation of biodegradable mulches increased the evaporation in the damaged parts. Future research should include in-depth and detailed analyses on the response mechanism between the amount of evaporation and the increase in precipitation infiltration considering the degradation of biodegradable mulches and the response relationship between precipitation utilization and soil moisture content before precipitation.

Previous studies on wheat, cotton, tomato, rapeseed, and other crops under drip irrigation with mulching showed that the yield under biodegradable mulching was lower than that under PM but with no significant difference, and the WUE values of the treatments were not significantly different. In this study, there was no significant difference between the yield and WUE under PM and biodegradable mulching with an induction period of 100 d. The highest yield in normal years was obtained under biodegradable mulching for the induction period of 60 d, and this yield was significantly higher than that under PM. On the one hand, in the middle and late stages of growth, after the degradable plastic film is damaged, the rainwater can directly infiltrate into the root zone of the crops, improving rainwater utilization. On the other hand, the heat preservation effect disappears due to damage of the film. The low temperature at night can slow down plants’ respiration, reducing unnecessary consumption, which is conducive to accumulating nutrients in plants and, thus, increasing production. In contrast, in the dry years, the lowest yield was obtained for the biodegradable mulching with an induction period of 60 d, and their yield and WUE were significantly lower than those under PM. The yield and WUE of biodegradable mulches with an induction period of 80 d were moderate during the 3-year experiment. Xu’s research on maize in northeast China revealed that mulching reduced yield and WUE in years with abundant precipitation, which supports the experimental results of this study [26]. However, Yin’s furrow irrigation experiment in Northwest China over 3 y showed that the highest yield of biodegradable mulches was obtained under a medium degradation rate, and this yield was significantly higher than that under PM and other biodegradable mulch treatments. Yin’s study also reported that although the total precipitation in 2014 was higher than in 2015, the yield and WUE in 2015 were higher than in 2014. Similar conclusions were obtained in this study, in which the yield and WUE of each treatment in 2016 were higher than those in 2017. The similarities and differences between the conclusion of this experiment and previous studies might be related to irrigation methods and regional climate. Therefore, according to different regions and types of hydrological years, different biodegradable mulches should be scientifically selected.

## 4. Materials and Methods

### 4.1. Field Experiments

The field experiments were conducted at the comprehensive technology demonstration base of drip irrigation under mulching (44°6${}^{\prime }$ N, 122°21${}^{\prime }$ E; 173.5 m above the sea level) in Tongliao, Inner Mongolian Autonomous Region, China, from 2016 to 2018. The site has a mean annual precipitation of 324 mm, mean annual evaporation of 2027 mm, mean annual groundwater table depth of 8.5 m, and mean annual sunshine duration of 2884.8 h. The soil physical properties and hydraulic parameters of the study area are shown in Table 2.

**Figure 5:**
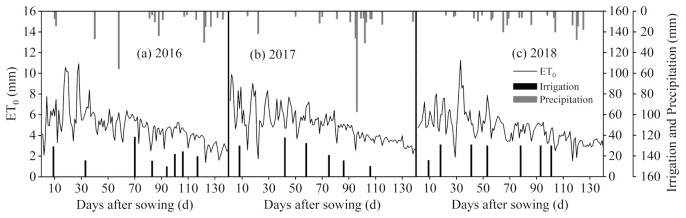
Irrigation, precipitation, and $ET_{0}$ in the growth period of 2016, 2017, and 2018. The amount of water applied to the field and the flow rate of the drip irrigation system of each treatment were monitored daily, separately, through independent water meters and valves. Precipitation data were obtained from an on-site weather station (HOBO U30 Onset).

A total of eight treatments were set up in the study, of which six were degradable mulch treatments: three white degradable mulch film treatments with an induction period of 60 d (WM60), 80 d (WM80), and 100 d (WM100); three black degradable mulch films with an induction period of 60 d (BM60), 80 d (BM80), 100 d (BM100). The other two treatments, ordinary plastic mulch (PM) and bare land (CK), were set as controls. The plot was 20 m long and 5 m wide, and each treatment was repeated three times. The ordinary plastic film and the biodegradable film were both 70 cm wide and 0.008 mm thick. The biodegradable film was provided by Shandong Tianzhuang Eco-Benign Plastics Technology Co., Ltd. (Jinan, Shandong Province, China). The planting model was “one mulch to one tube to two rows” (Figure 1). Eccentric sowing was conducted with a small row spacing of 35 cm, row width of 85 cm, distance between adjacent drip irrigation belts of 1.2 m, plant spacing of 24.6 cm, and planting density of 67,500 plants per ha. The amount of irrigation water in this experiment was set according to the percentage of soil moisture content in the field water capacity: 65–85% during sowing to jointing period; 75–95% during jointing to grouting period; 70–85% during grouting to maturity period. The actual irrigation amount is designed according to the measured soil moisture content of PM treatment. Maize was irrigated with a drip irrigation system, with a dripper spacing of 25 cm, and the flow rate was 1.8 L/h. A complete set of water meters and valves were separately installed for each treatment to monitor the amount of water applied to the field and the flow rate of the drip irrigation system (Figure 5). The variety of spring maize was Nonghua 106, which has a growth period of 129 d. The precipitation during the growing seasons of 2016, 2017, and 2018 was 272.03, 290.42, and 212.56 mm, respectively. The years 2016 and 2017 were considered to have a normal flow, whereas 2018 was considered a low-flow year [27]. Conventional methods were adopted for fertilization; supporting measures of agricultural machinery and agronomy; and management of diseases, pests, and weeds. The maize crops were planted on 29 April 2016, 27 April 2017, and 27 April 2018, and they were harvested on 26 September 2016, 24 September 2017, and 25 September 2018, respectively.

### 4.2. Field Climate Data

Field climate data were obtained from a weather station (HOBO U30 Onset, Onset Computer Corporation, Bourne, MA, USA) installed at the experimental site. Reference crop evapotranspiration ($ET_{0}$) was calculated using the Penman–Monteith equation (Figure 5).

To investigate the rainwater utilization under different precipitation values and different degradation rates of biodegradable mulches, we selected eight precipitation events for analysis according to the precipitation data for the test area from 2016 to 2018 and the precipitation intensity classification standard (inland part) issued by the China Meteorological Administration (Table 3).

### 4.3. Soil Moisture Content

The soil moisture content was monitored using a time-domain reflectometer (TDR, TRIME-PICO-IPH, Germany) tube and an automatic soil moisture recorder (THL-TWS-18, produced by Beijing Kunlun Taiheng Technology Co., Ltd., Beijing, China). The results were verified using the gravimetric method. The TDR soil moisture meter was used to measure soil moisture content every 10 d after sowing. Additional measurements were conducted before irrigation, after irrigation, and after precipitation. The sampling position was directly below the drip irrigation tape between narrow rows (r = 0 from the drip irrigation tape), between rows of maize (r = 17.5 cm), at the edge of the mulches (r = 35 cm), and between wide rows (r = 60 cm). The samples were collected from soil depths of 0–20, 20–40, 40–60, 60–80, and 80–100 cm.

For the PM, WM60, BM60, and CK treatments, in addition to the gravimetric method and TDR tube to collect soil moisture, the automatic soil moisture recorder was used to monitor the soil moisture content during the growth period. Data were collected every 1 h, and the monitoring points were directly below the drip irrigation belt, between rows of maize, and between wide rows at soil depths of 10, 30, 50, 70, and 90 cm.

### 4.4. Degradation Rate of the Biodegradable Mulches

The degradation rate of the biodegradable mulches gradually decreased along the vertical direction of the drip irrigation belt, and the mulching area was divided into Zone I, Zone II, and Zone III (Figure 1). The mulches in Zone I were exposed on the ground surface, and after sowing, three observation points within the plot were randomly selected and then delineated and marked with a wooden frame. The area inside the frame was 38 cm × 38 cm, and three photos were obtained every 10 d. The photos were imported into CAD, the damage boundary was drawn, and the area of damage in Zone I was statistically calculated. The degradation rate ($FD_{1}$) was calculated as follows:

(2) $${FD}_{1}=\frac{\sum_{i=1}^{n}A_{1}^{{}^{\prime }}}{A_{1}}$$

where $A_{1}$ is the total area of Zone I (cm ${}^{2}$), $A_{1}^{{}^{\prime }}$ is the damaged area (cm ${}^{2}$) of biodegradable mulches in Zone I, and *i* has a value of 1–n.

Zones II and III are covered by thin soil layers; therefore, it is impossible to take photos continuously to record the extent of degradation. Therefore, the damaged area was estimated according to the weight loss of the mulch film. First, before seeding, the six degradable mulching films were weighed at 0.1 m × 1 m, respectively; this was repeated three times, and the average value was denoted as $M_{0}$. Every ten days after sowing, three 1 m long mulch sections were randomly selected in each plot, and the mulch film was collected from the different areas and brought back to the laboratory to be cleaned with an ultrasonic cleaner; after natural air-drying, the samples were weighed using a balance with ten-thousandth precision. Mulch film in Zones 2 and 3 were recorded as $M_{2}$ and $M_{3}$, respectively, and the damage rates of the plastic film in each zone were calculated separately. The calculation formulae are as follows:

(3) $$FD_{2}=\frac{M_{0}-\frac{M_{2}}{1.5}}{M_{0}}$$

(4) $$FD_{3}=\frac{M_{0}-M_{3}}{M_{0}}$$

where $M_{0}$ is the undegraded mulch film (*g*), $M_{2}$ is the degraded mulch film from Zone II (*g*), and $M_{3}$ is the degraded mulch film from Zone III (*g*).

The total degradation rate (average fraction of the degradation area, *AFD*) of biodegradable mulches was calculated as follows:

(5) $$AFD=\frac{{FD}_{1}\times 10+{FD}_{2}\times 15+{FD}_{3}\times 10}{35}\times 100\%$$

### 4.5. Precipitation Effective Infiltration Ratio

Under the planting mode of this study, the corn root system is mainly distributed within the range of 0–35 cm away from the drip irrigation belt, which is also the film-covered area. The difference between the soil water storage in the 0–100 cm soil layer before and after rain in this area is recorded as the precipitation effective infiltration amount, denoted as $P_{e}$. The soil moisture content chooses the average value of 4 h before and after rain, respectively. The ratio of precipitation effective infiltration amount to rainfall is recorded as precipitation effective infiltration ratio λ. The detailed calculation formula is as follows:

(6) $$\lambda =\frac{P_{e}}{P}$$

where λ is the precipitation effective infiltration ratio, %; $P_{e}$ is the precipitation accumulated in the area under mulching (0 to 35 cm from the drip irrigation belt), mm; and *P* is the total precipitation each time, mm. To measure the yield, three 10 m long double rows of maize were selected parallel to the drip irrigation belt in each plot, and all grains were harvested and air-dried to derive their total weight, which was converted into the yield per hectare.

SigmaPlot 18 was used to fit precipitation data, biodegradable mulch degradation rate, and effective infiltration ratio of precipitation, and multiple linear regression was used to obtain the model equation of precipitation and mulch degradation rate.

### 4.6. $ET_{c}$ and WUE

In calculating water balance [28] and WUE, groundwater recharge was ignored because the groundwater depth measured locally was 8.5 m. Furthermore, the surface runoff and deep seepage were ignored because the irrigation method was drip irrigation and the surface was flat.

(7) $$ET_{c}=I+P+\Delta W$$

(8) $$WUE=\frac{Y}{ET_{c}}$$

where $ET_{c}$ is the total water consumed by evapotranspiration (mm); *I* and *P* are irrigation water applied (mm) and effective precipitation (mm), respectively; Δ*W* is the water storage difference (mm) during the calculation of $ET_{c}$; *WUE* is the water use efficiency (kg /mm·ha); and *Y* is yield (kg /ha).

### 4.7. Data Statistics and Analysis

Excel 2016 was used to organize the basic data. Analysis of variance (ANOVA) was performed using the general linear model in Origin 2018 to test the effects of mulch type on yield and WUE. Differences were determined using Duncan’s multiple range test at the 5% level of significance. Pearson’s correlation coefficients were used to analyze the relationships among the precipitation effective infiltration ratios of the three types of mulches.

## 5. Conclusions

The precipitation effective infiltration ratio was affected by precipitation to a larger extent than the degradation rate. As precipitation increased, the precipitation effective infiltration first decreased and then increased. When cumulative precipitation reached 89.21 mm, mulching no longer affected precipitation utilization. As the degradation rate of biodegradable mulches increased, the precipitation effective infiltration ratio also gradually increased, but the magnitude of this increase gradually decreased with the increase in degradation rate. After the degradation rate of the biodegradable mulches reached 36.64%, the impact of precipitation utilization was no longer significant.

In normal years, the highest yield and WUE were obtained for biodegradable mulches with an induction period of 60 d, whereas in dry years, they were obtained for biodegradable mulches with an induction period of 100 d.

For maize planting under drip irrigation with mulching in the West Liaohe Plain, a biodegradable mulch with an induction period of approximately 60 d is recommended for normal years with a degradation rate of 36.64%, whereas a biodegradable mulch with an induction period of 100 d is recommended for dry years.

## Figures and Tables

**Table 1:** Effects of different film mulching on yield and water use efficiency (WUE) of maize.

Year	Treatment	ΔW (mm)	${\mathrm{ET}}_{c}$ (mm)	Yield (kg/ha)	WUE (kg/mm·ha)
2016	PM	−20.89	428.68b	12,271.20b	28.63ab
	WM60	1.12	450.69ab	13,484.11a	29.92a
	WM80	−4.05	445.52ab	12,994.78ab	29.17a
	WM100	−14.18	435.39b	12,471.34b	28.64ab
	BM60	2.73	452.3ab	13,222.36a	29.23a
	BM80	−2.41	447.16ab	12,937.15ab	28.93a
	BM100	−13.77	435.79b	12,335.47b	28.31b
	CK	5.94	455.51a	12,922.25ab	28.37b
2017	PM	−13.95	459.79a	11,686.8c	25.41c
	WM60	−2.39	471.35a	13,379.01a	28.38a
	WM80	−3.32	470.43a	12,848.67ab	27.31ab
	WM100	−8.78	464.97a	12,727.16ab	27.37ab
	BM60	−1.92	471.83a	12,813.34ab	27.15ab
	BM80	−2.52	471.22a	12,287.64b	26.07b
	BM100	−7.64	466.11a	12,277.16b	26.33b
	CK	3.37	477.11a	12,238.95b	25.65b
2018	PM	−2.76	419.62a	12,075.62b	28.77ab
	WM60	13.56	435.95a	11,193.13c	25.67c
	WM80	9.24	431.62a	12,461.2ab	28.87ab
	WM100	0.81	423.19a	12,732.29a	30.08a
	BM60	14.92	437.30a	11,238.01c	25.69c
	BM80	10.62	433.01a	12,053.27b	27.83b
	BM100	1.05	423.43a	12,256.73ab	28.94ab
	CK	15.65	438.04a	10,526.10d	24.02d

Different lowercase letters in the same column indicate significance (*p* < 0.05).

**Table 2:** Soil particle composition and parameters of soil hydraulic property.

Soil Layers (cm)	Soil Separates (%)			Bulk Density (g/cm ${}^{3}$)	Field Capacity (cm ${}^{3}$/cm ${}^{3}$)	Wilting Point (cm ${}^{3}$/cm ${}^{3}$)	Saturated Water Content (cm ${}^{3}$/cm ${}^{3}$)
	Sand	Silt	Clay				
0–20	36.76	52.70	10.54	1.39	0.26	0.075	0.403
20–40	21.65	48.81	29.54	1.38	0.30	0.079	0.451
40–70	20.18	39.15	40.67	1.41	0.46	0.098	0.532
70–100	77.08	21.65	1.27	1.52	0.18	0.052	0.378

Notes: wilting point refers to the soil water content that cannot be absorbed and utilized by plants.

**Table 3:** Precipitation and precipitation classification at different times.

Date	2016			2017		2018		
	28 June	27 July	30 August	7 July	23 August	4 July	25 July	11 August
precipitation level	EHP	HP	EHP	MP	MP	HP	MP	HP
precipitation (mm)	54.6	23.6	45.8	11.6	12.8	20	13	24.6
precipitation duration (h)	5	4	11	4	4	12	2	11

Notes: MP, moderate precipitation; HP, heavy precipitation; EHP, extremely heavy precipitation.

## Data Availability

Not applicable.

## Abbreviations

The following abbreviations are used in this manuscript:

WM	white biodegradable mulch
BM	black biodegradable mulch
FD	fraction of the degradation area
AFD	average fraction of the degradation area
TDR	time-domain reflectometry
WUE	water use efficiency
